# Mandibular reconstruction with anterolateral thigh free flap and bridging plate: a retrospective study of 34 oncological cases

**DOI:** 10.3389/fsurg.2025.1610229

**Published:** 2025-06-05

**Authors:** Caterina Ottaviano, Funda Goker, Marcello Cammalleri, Andrea Fior, Vittorio Favero, Tommaso Rizzo, Guido Lobbia, Gianluca Colapinto, Massimo Albanese, Massimo Del Fabbro, Giorgio Barbera

**Affiliations:** ^1^ Section of Oral and Maxillofacial Surgery, University of Verona, Verona, Italy; ^2^ Policlinico Giovanni Battista Rossi, University of Verona, Verona, Italy; ^3^ Department of Biomedical, Surgical and Dental Sciences, University of Milan, Milano, Italy; ^4^ Unit of Maxillofacial Surgery and Dentistry, Fondazione IRCCS Ca’ Granda Ospedale Maggiore Policlinico, Milano, Italy; ^5^ Department of Oral and Maxillofacial Surgery, Faculty of Dentistry, Istanbul Aydın University, Istanbul, Turkey; ^6^ Section of Oral and Maxillofacial Surgery, Policlinico Universitario, University of Padova, Padova, Italy

**Keywords:** ALT flap, custom-made prosthesis, mandibular reconstruction, oral cancer, reconstruction plate

## Abstract

**Introduction:**

The surgical gold standard following mandibular resection is reconstruction with a vascular osteocutaneous free flap. However, not all patients are suitable candidates for this type of procedure due to local or systemic contraindications or the critical anatomy of the remaining healthy bone. This study aimed to evaluate the outcomes of reconstruction with an anterolateral thigh (ALT) free flap in combination with a reconstruction plate as an alternative in such situations.

**Methods:**

This retrospective case series included 34 oncological patients treated with ALT and reconstruction plate to restore mandibular bone defect following mandibulectomy. Seven patients who experienced serious postoperative complications received additional surgery involving the replacement of titanium plates [three with custom-made plates using computer-aided design (CAD)/computer-aided manufacturing (CAM) methods]. For evaluation, the following data were collected from the patients: gender, smoking habits, information about surgery (such as type of demolition, type of reconstruction plate used, and duration), the pathological node involvement, the application of radiotherapy (pre- or postoperative), the onset of complications (type of complication, time to complication, management strategy), and follow-up data. The follow-up period of the patients ranged from 3 months to 7 years. The follow-up assessments were based on both clinical and radiological methods, with CT scans performed at 1, 3, and 6 months.

**Results:**

Thirteen out of 34 patients developed early or late complications and were candidates for reoperation. Six of them died before undergoing additional surgery. The complications that were observed included flap failure, complications related to the plate (exposure, fracture, dislocation), development of sarco-radionecrosis, and recurrence. Seven subjects received additional surgery, including the removal and replacement of the reconstruction plates (custom-made plates in three patients).

**Conclusion:**

The surgical protocol described in this paper represents a promising option mostly suitable for geriatric patients who show relatively poor health status and advanced tumor stages.

## 1 Introduction

Oral cancer represents one of the most common head and neck cancers in Europe (1). The management protocol adopted in many centers involves primary ablative surgery followed by radiotherapy and chemotherapy in selected cases. Many centers perform one-stage reconstruction surgery, while some surgeons prefer two-stage surgeries (2). These types of patients usually require reconstruction which is challenging, especially in the case of composite and extensive oromandibular defects resulting from excision of Stage 4 (T4 as it refers to advanced stage) cancers (3). Rehabilitation options for mandibular defects depend on numerous factors including the extent and location of the defect, the patient's general health condition, prognosis, the patient's choice, and the surgeon's experience (3–6). A single osteocutaneous flap is the most preferred option for most of these defects; however, in some cases, it may be necessary to use an additional free flap (3).

Currently, bony reconstruction using vascularized flaps nevertheless remains the gold standard; in this regard, the free fibula flap offers a versatile approach with multiple techniques described in the literature. As an additional advantage, a free fibula flap can allow a bilateral reconstruction of the mandibular body using a single fibula (7–10). In some cases, a simpler method can be preferred which involves the use of a titanium plate to bridge the bone defect together with a vascularized fasciocutaneous or myocutaneous flap for soft tissue reconstruction (3, 6, 11–13). However, the latter method can represent a higher risk of complications, including dehiscence of the overlying soft tissue with plate exposure, plate dislocation, loosening of the screws, and/or fractures of the plate (13–20). Various studies have shown that these complications are more frequent in the anterior area of the jaw and their incidence is directly related to the size of the defect (6, 12, 13). In cases of large soft tissue defects, anterolateral thigh (ALT) flaps offer some advantages as they can be harvested as fasciocutanous or myocutaneous and can be custom-designed to fit the defect in the maxillofacial region (14). ALT free flap is a soft tissue flap that is characterized by faster inset, shorter operative times, vessels less damaged by vascular pathologic conditions, lower comorbidity at the donor site, and reduced bed rest for the patient when compared with other vascularized free flap alternatives. Nevertheless, the decision to utilize titanium reconstruction plates in combination with ALT flap should depend on the health condition and the cancer stage of the patient (15, 16). Therefore, this type of surgery is considered under very rare conditions. Although not frequent, there is always a risk for complications, and management is challenging. In the literature, the reports on management methods for comorbidities associated with maxillary reconstruction using vascularized ALT flap with a reconstruction plate are very limited. For this reason, this study aimed to present the results of rehabilitation of oral cancer patients with large mandibular defects (with critical size and anatomy), compromised health status, and advanced age by surgical reconstruction operation using ALT flaps and bridging titanium reconstruction plates and to describe how to handle complications that might be associated.

## 2 Materials and methods

This retrospective case series study was conducted at the University of Verona which has a joint agreement with the University of Milano for scientific research. The treatment protocol followed the principles laid down in the Declaration of Helsinki on medical protocol and ethics. The study protocol was approved by the Ethics Committee of Fondazione IRCCS Ca' Granda Ospedale Maggiore Policlinico, Regione Lombardia, with the 21 February 2017 Ethics Committee of Milano Area B Act 478/2017.

Between 2016 and 2023 at the Maxillofacial Surgery Unit of the University of Verona, 175 patients underwent demolition and reconstruction surgery for carcinoma of the oral cavity involving the mandibular bone. Following mandibular resections, 141 cases were rehabilitated with free fibula flaps or scapula flaps. The other 34 patients received reconstructions with bridging titanium reconstruction plates and soft tissue ALT free flaps.

In this study, the classification created by Della Monaca–Valentini (20) was used to identify jaw resection type. The classification is as follows: **B1**, symphysis resection between two mental foramina; **B2**, deficits involving the unilateral mandibular body and partial ramus and not extending beyond the symphysis; **B3**, unilateral defects with posterior ramus involvement but not extending beyond the symphysis; **B4**, isolated mandibular ramus defects; **B5**, defects that exceed the symphysis and extend beyond the contralateral parasymphysis.

The inclusion criteria were as follows: patients aged ≥70 years at the time of surgery, those with oral cancer stage IV (T4) (21), those with comorbidities (such as diabetes mellitus, elevated blood pressure, hypercholesterolemia, systemic vasculopathy, peripheral vascular disease involving the lower extremities, hypoplastic anterior tibial artery, small skin paddle available with bone flaps, venous insufficiency, deep vein thrombosis, history of contralateral lower extremity amputation), and those classified as ASA class III or IV. Patients who had received reconstructions with a titanium reconstruction plate and soft tissue anterolateral thigh (ALT) free flap after mandibular resections were included. The exclusion criteria for the study were patients with stages lower than T4 and those operated with free flaps other than ALT flaps.

### 2.1 Surgical protocol

**Figure 1:**
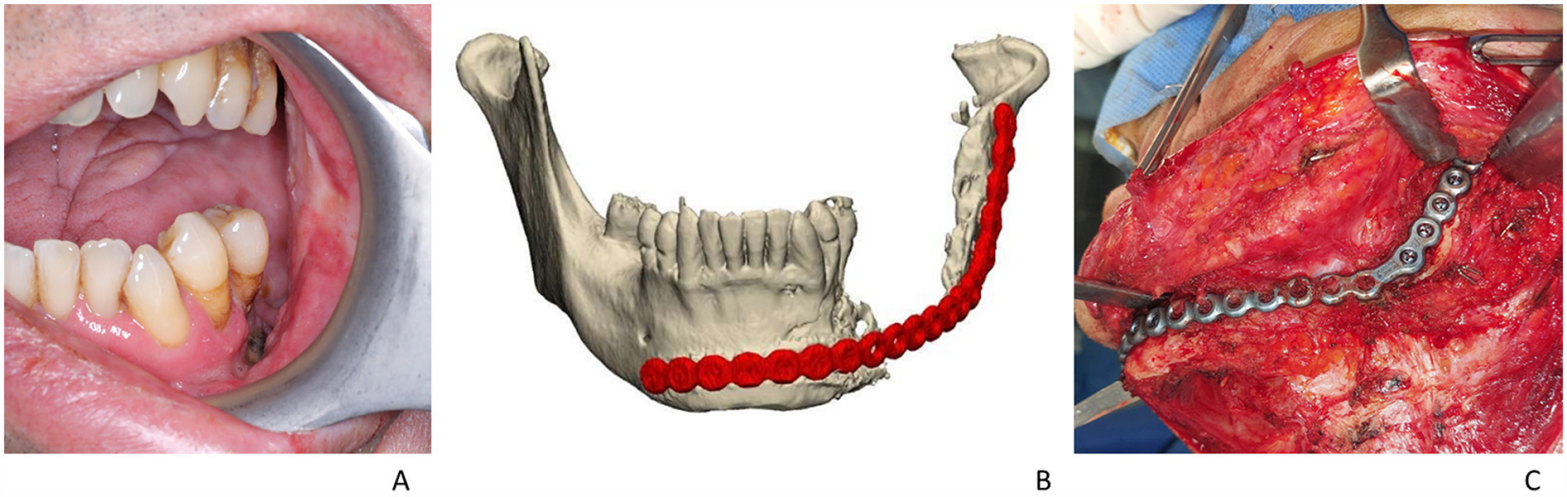
**(A)** Intraoral view showing sarco-radionecrosis; **(B)** preoperative planning stage—the previous 2.4 titanium plate used to restore the bone gap; **(C)** intraoperative image showing placement of 2.4 reconstruction plate before removal.

**Figure 2:**
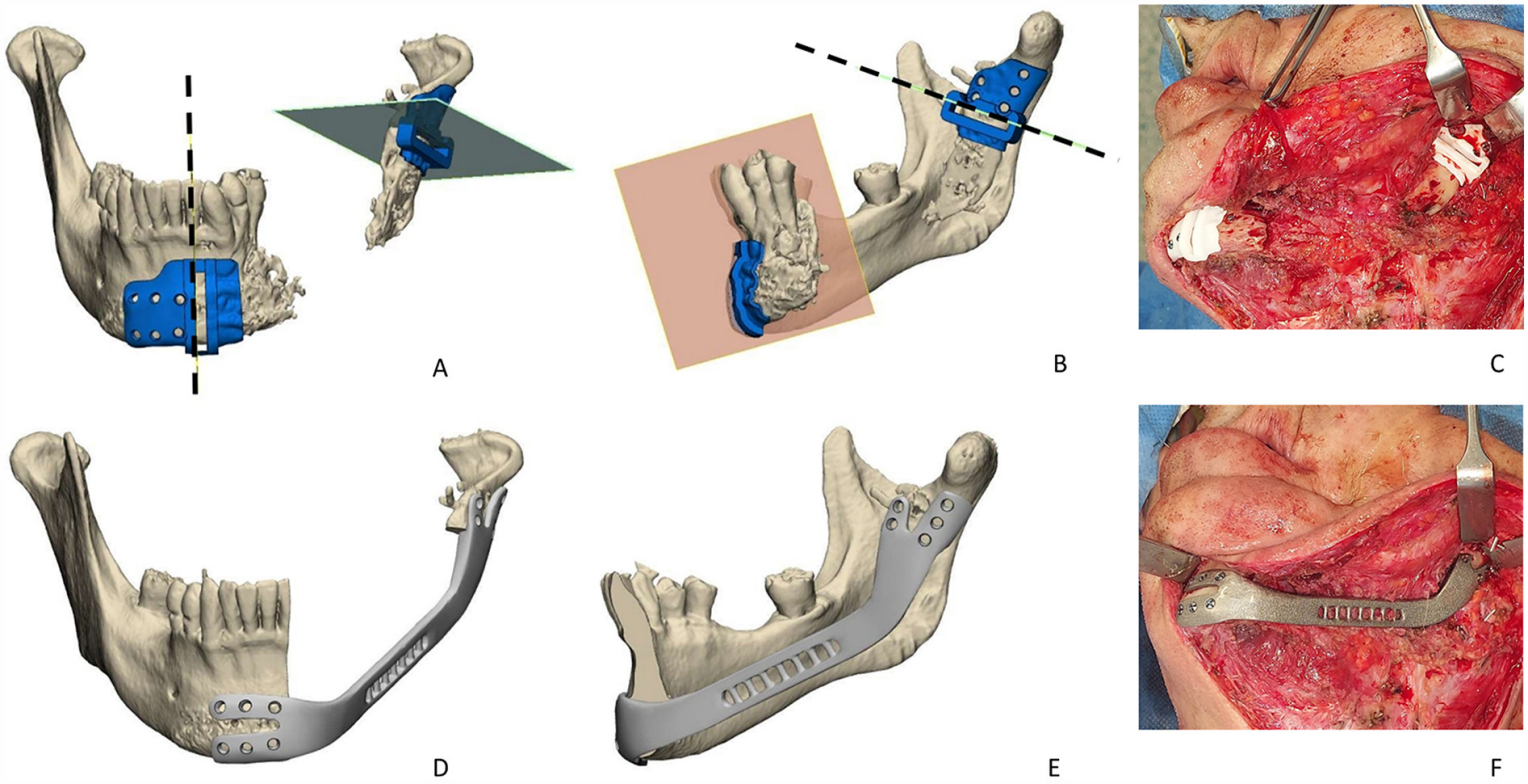
Planning and reconstruction of the same patient that had sarco-radionecrosis. Following the removal of the plate, it was replaced with custom-made reconstruction plate: **(A)** right cutting guide, **(B)** left cutting guide, **(C)** intraoperative image showing cutting guides in place, **(D)** planning of custom-made plate frontal view, **(E)** custom-made plate lateral view, **(F)** intraoperative image showing custom-made plate fixed on the mandible.

All surgical procedures were performed in the same session under general anesthesia. Following hemimandibulectomy (HM), the bone defect was rehabilitated using 2.4 mm (24 cases) or 2.5-mm-thick (10 cases) bridging reconstruction locking titanium plates fixed with locking screws. In all cases, the reconstruction plate was bent accordingly before mandibular resection. The mandibular bone was resected, and the plate was fixed on the remaining mandible. Soft tissue defect was covered using a vascularized ALT free flap. The ALT flap was prepared as a fasciomyocutaneous flap or fasciomuscular flap, depending on the cases, due to neoplastic infiltration of the skin. The flap shape and width were defined with a sterile gauze template, contoured according to the dimensions of the defect and replicating its shape. Out of 34 patients, seven patients had severe complications and needed second surgery for management which included replacement of reconstruction plates. Three of these patients had custom-made (patient-specific) plates that were produced using computer-aided design (CAD)/computer-aided manufacturing (CAM) methods. Figures 1 and 2 show the representative photos of one of these three patients who had received additional surgery to remove of reconstruction plate that was replaced by patient-specific custom-made plate.

#### 2.1.1 Custom-made plate

The custom-made plates were preferred in cases of severe complications such as plate fracture, plate dislocation, or sarco-radionecrosis, and a CT scan was the first step in planning. The decision-making process for choosing the customized plate depended on the fact that it adapts better to the residual bone stumps and is less bulky than the standard plate. Furthermore, with the standard plate microgaps can be present between the plate and the bone surface, which creates tension on the fixing screws with a higher possibility of screw loss or non-osseointegration that can cause failure. The greater dimensions create the conditions for greater pressure on the tissues associated with possible ischemic problems which in return can cause dehiscence of the plaque and infections.

Custom-made plates used in this study were titanium Ti6Al4V ELI alloy made by the Mt Ortho Srl. (Catania, Italy) using electron beam melting (EBM) technology with a fabrication method based on computer tomography images of the patients and were manufactured within 2 weeks. The company received instructions from the surgeon to develop a design that included a double anchoring arm and a shoulder profile for the retention structures on both abutments with a less convex profile when compared with the mandibula. In cases where it is also necessary to restore a soft tissue deficit, the plate was designed with a reduced profile compared with the original one, to avoid excessive bulging due to the restoration of the soft tissue itself with a musculocutaneous or fasciocutaneous flap. In Figure 1, the preoperative stage of one of the patients that had severe complications can be seen (the patient had a reconstruction plate which was removed to be placed with a custom-made plate. Figure 1A, preoperative intraoral view; Figure 1B, 3D model of the custom-made plate was created using CT images; Figure 1C, the intraoperative image showing 2.4 reconstruction plate before removal). The surgical protocol continued with the removal of the plate causing the complication via a cervicectomy approach. The removed plate was subjected to microbiological and culture analysis. In cases where the patient presented osteoradionecrosis, the bone stumps were resected using two cutting guides, one positioned at the mandibular symphysis and the other at the condylar process. The resected bone segments were sent for histological examination. The cutting guides were designed with a perforated pattern that facilitated drilling execution for the placement and stabilization of customized plates and screws (Figures 2A–C). The custom-made plate was positioned and fixed with titanium screws following the application of rigid intermaxillary fixation (Figures 2D–F). Once the intermaxillary fixation was removed, the occlusion was checked to ensure it was satisfactory. In cases of sarco-radionecrosis, a pedicled pectoralis major flap was preferred to fill the soft tissue defect and provide appropriate coverage. The custom-made plate consisted of two retaining structures at the interfaces with the bone surfaces. A grid perforated structure can be observed along the lateral profile of the plate (Figures 2E,F), which was designed to facilitate the reattachment of the muscles and of the other soft tissues that are disconnected from their original site of insertion.

In Table 1, a comparison of custom-made and regular reconstruction plates is reported for further information.

**Table 1:** Mechanical properties of custom-made prosthesis and 2.4 reconstruction plate.

Mechanical properties of materials							
Device	Material	Young's modulus (Gpa)	Poissons's ratio	Friction coefficient	Yield strength (Mpa)	Tensile strength (Mpa)	Endurance limit (Mpa)
2.4 reconstruction plate	Titanium Grade 2	105	0.35	0.3	276	345	173
Custom-made prosthesis Mt Ortho	Ti6Al4V ELI	120	0.3	0.3	930	970	600

### 2.2 Data collection

The following information was collected from the patients: gender, smoke habits, details about surgery (such as type of demolition, type of reconstruction plate used, duration), the pathological node involvement, the use of radiotherapy (pre- or postoperative), the onset of complications (type of complication, time to complication, management strategy), and follow-up data. Complications were divided into early complications (within 1 week or less) or late complications (after >1 week). The complications we analyzed were as follows: flap failure, complications related to the plate (exposure, fracture, dislocation), development of sarco-radionecrosis, and disease recurrence. The complication evaluation protocol was both clinical and radiological. Follow-up of the alt flap was performed every 2 h on the first day and then daily for 1 week, with subsequent CT. The follow-up period of the patients ranged from 3 months to 6 years. The follow-up was both clinical and radiological, with a CT scan performed 1 month and 3 months after the procedure. The patient follow-up protocol with custom-made plates: Patients were checked 1 week, 1 month, 6 months, and 1 year after the second surgery. Patients had a CT scan immediately postoperatively and at a follow-up 6 months after surgery. The conditions that might require the replacement of the standard plate included surgical plate exposure, infection associated with the development of osteonecrosis, osteomyelitis, plate dislocation or fracture or detachment from the bone, or loosening/loss of the fixing screws. The procedure or salvage surgery was considered successful in cases of stability at 1 month with the healing of the soft tissues around the new reconstruction plate, with the plate firmly in place, and in the absence of fistulas, exposures, loosening of the fixing screws which were confirmed with clinical–radiological evaluations.

### 2.3 Statistical evaluation of data

In this study, only descriptive statistics were made for the evaluation of data. No comparative statistics were performed due to the heterogeneity of patient condition and treatment received. Patients' demographics were presented using mean values ± 1 standard deviation (SD) and percentages, calculated using Microsoft Excel.

## 3 Results

Thirty-four patients (23 male/11 female) were included in the study. The mean age of the participants was 74 ± 4 years. The oncological conditions were 30 squamous cell carcinomas, 3 sarcomatoid carcinomas, and 1 adenoid cystic carcinoma. All cases were R0 according to the residual tumor classification (22). Hemimandibulectomy was performed in 16 cases (B3), partial mandibulectomy in 11 cases (B2), segmental mandibulectomy of the ramus in 4 cases (B4), and segmental anterior mandibulectomy in 3 cases (B1) (20). Recurrence of disease occurred in 14/34 patients (41%). Thirteen out of 34 patients developed early or late complications (38%) and were candidates for reoperation. Six of these deceased before additional surgery. The complications observed were as follows: flap failure, complications related to the plate (exposure, fracture, dislocation), development of sarco-radionecrosis, and disease recurrence. Out of these, seven subjects received additional surgeries, including removal of the reconstruction plates which were replaced with custom-made plates that were produced using CAD/CAM methods in three patients.

Table 2 shows the patient characteristics (gender, smoke habitus), surgical information (type of demolition, type of reconstruction plate used, duration), the pathological node involvement grade (N), the use of chemotherapy/radiotherapy (pre- or postoperative), the onset of complications (type of complication, time to complication, second treatment strategy), and follow-up. The mean follow-up duration was 26 months (from 3 to 84 months). A follow-up of such short duration (3 months) is justified by the fact that some patients deceased due to worsening of their health condition after a few months, given their advanced oncological condition and rapidly deteriorating clinical status.

**Table 2:** Case series data.

Gender	Smoking habit	Classification of surgery (20)	Time (min)	N	Radiotherapy			Complications (time to complications)	Second treatment	Recurrence (time to recurrence)	Follow-up
					Pre	Post					
M	No	B1	735	N1	–		Yes C + Rx	Flap necrosis Early complication (4 days)	2.4 P + ALT flap	–	1.5 years, deceased
M	Yes	B1	530	N1	–		Yes	–	Palliation	Local and nodal recurrence (7 months)	1 year, deceased
M	No	B1	753	N2b	–		–	Plate exposure (6 months)	Plate removal	–	2 years, deceased
F	No	B2	585	N0	–		–	Plate dislocation (6 years)	Placement of custom-made prosthesis	–	6 years
M	Yes	B2	712	N0	Yes Rx + C		–	–	Palliation	Local recurrence (1 year) + C	4 years, deceased
M	No	B2	480	N0	–		Yes	Necrotic bone and plate exposition, submental fistula (9 months)	Mandibular osteotomy + Rx	Local recurrence and distant metastasis (9 months)	2 years, deceased
F	No	B2	352	N0	–		–	Plate fracture (3.5 years) Second plate fracture (14 months)	2.4 P + CmP	–	6 years
M	Yes	B2	600	N1	–		Yes	Flap necrosis Early complication (4 days)	Latissimus dorsi flap	–	4 years
M	Yes	B2, partial maxillectomy	620	N1	–		Yes	Plate superinfection, sarco-radionecrosis (11 months)	CmP + pectoralis major flap	–	3 years
F	No	B2	572	N2	–		Yes	–	–	–	10 months, deceased
M	No	B2	770	N2	–		-	–	–	–	1 year, deceased
M	Yes	B2	801	N2b	–		Yes	–	Palliation	Distant metastasis (8 months)	10 months, deceased
M	Yes	B2	780	N3b	–		Yes	–	–	–	3 years
M	No	B2	–	N3b	–		Yes C + Rx	–	Contralateral HM + fibula flap	Local recurrence, Sc (2 years)	3 years
M	Yes	B2	597	N3b	–		Yes	–	–	–	1 year
M	No	B3	765	N0	–		–	–	–	–	3 months, deceased
F	No	B3	526	N0	–		Yes	Fistulation, plate exposure, and plate fracture (12 months)	Removal of the plate	Local recurrence (12 months)	1 year, deceased
M	Yes	B3 (Sc)	600	N0	Yes		–	–	–	–	1 year, deceased
F	No	B3 (Sc)	435	N0	–		– C	–	–	–	1 year, deceased
F	Yes	B3	694	N0	–		–	(1) Flap necrosis (5 days) (2) Submental plate exposure (10 months)	(1) ALT flap (2) Palliative therapy	–	1 year, deceased
M	No	B3	538	N0	Yes		–	–	–	Local recurrence of basocellular carcinoma	3 years, alive
F	No	B3	590	N0	–		–	–	–		–
M	Yes	B3	595	N0	–		–	–	–		2 years
F	No	B3	523	N0	–		–	–	Palliation	Local recurrence 4 months	4 months, deceased
F	No	B3	712	N1	–		Yes	Partial flap necrosis Early complication (4 days)	Radial flap	Local recurrence with cutaneous fistula (5 months)	11 months, deceased
M	Yes	B3	630	N2b	–		Yes C + Rx	–	C + Rx	Local and nodal recurrence (6 months)	2 years, deceased
F	No	B3 Partial GS (Sc)	720	N2	–		–	–	–	–	1 month, deceased
M	Yes	B3	633	N2a	–		–	Plate fracture (2.5 years)	2.5 P + palliation	Local recurrence (6 years)	6 years
M	Yes	B3	631	N2b	–		–	Plate exposure (4 months)	Cytoreductive CT	Local recurrence (4 months)	1 year, deceased
M	–	B3	440	N3b	–		–	–	Palliation	Distant metastasis (lung carcinoma- 1 month)	1 month, deceased
F	No	B3	650	N3b	–		–	–	Palliation	Nodal recurrence	4 months, deceased
M	No	B2	–	N3b	–		Yes C + Rx	–	Contralateral HM + fibula flap	Local recurrence, Sc (2 years)	3 years
M	No	B3	771	N3b	–		Yes	–	–	–	10 months
M	No	B4	547	N0	–		-	Flap necrosis Early complication (3 days)	ALT flap	–	7 years
M	No	B4	728	N0	–		Yes	–	–	–	4 years, deceased

F, female; M, male; M: B1, B2, B3, B4, resection type classification created by Della Monaca–Valentini (20); N; N0, N1, N2a–b, N3b, pathological node involvement grade; C, chemotherapy; Rx, radiotherapy; Cytoreductive CT, cytoreductive surgery with the guidance of computed tomography; P, reconstruction plate placement; HM, hemimandibulectomy; ALT, anterolateral thigh flap; Sc, squamous cell carcinoma; CmP, custom-made plate; GS, glossectomy.

The overall mean duration of the surgical intervention was 625 min (range, 352–801 min). This included all the main phases of the operation, ablative surgery associated with lymphadenectomy of the lymph node compartments of the neck, and reconstruction. The reconstruction phase duration, which involved the preparation of the flap, isolation of the pedicle and its liberalization, and the insetting with microsurgical anastomoses, averaged approximately 2 h and 30 min.

Complications occurred in 13 patients. Of these, four patients had more than one complication. Tables 2 and 3 report the details of complications, recurrence, and treatments. Early complications included flap failure that occurred in five patients (15%) because of ischemia or microvascular circulation problems. In these cases, already in the first postoperative week, there was dehiscence of the flap. To solve the complication, secondary surgery involved the use of an alternative soft tissue flap after the removal of the first one: radial forearm free flap (one case), contralateral ALT free flap (three cases), latissimus dorsi free flap (one case). Late complications were various: plate exposure (five cases), sarco-radionecrosis (two cases), plate superinfection (one case), plate fracture (four cases), plate dislocation (one case). Plate exposure occurred in five cases. The average time of plate exposure was approximately 8 months. Two of them received radiotherapy after surgery. Details of patients that received secondary surgery: plate removal only (three cases), plate removal associated with mandibular necrotic bone removal, and first intention closure (one case) or palliative therapy (one case). In these cases, given the patient's poor clinical condition, a minimally invasive approach was considered the most appropriate intervention option.

**Table 3:** Mandibular reconstruction with custom-made plate and ALT free flap: complications, recurrence, and treatments.

Complication type	Number of cases (%)	Radiotherapy (%)		Mean time to complication	Treatment	
		Neoadjuvant	Adjuvant			
Early complications						
Flap necrosis	5		3	3–4 days	Plate removal, placement of plate 2.4	20%
					Flap removal, latissimus dorsi flap	20%
					ALT flap, palliative therapy	20%
					Necrotic flap removal, radial flap plate	20%
					ALT flap	20%
Late complications						
Flap failure	5	–	3	1 month	Radial flap	20%
					Contralateral ALT flap	40%
					Necrotic flap removal, plate substitution, contralateral ALT flap	20%
					Flap removal, latissimus dorsi flap	20%
Plate exposure	5	–	2	8 months	Plate removal only	60%
					Plate removal, mandibular osteotomy, first intention closure	20%
					Palliative therapy	20%
Sarco-radionecrosis	2	–	2	10 months	Plate removal, resection of the necrotic bone, first intention closure	50%
					Plate removal, resection of the necrotic bone, custom-made prosthesis positioning, pectoralis major flap	50%
Plate fracture	4*	–	1	26 months	Plate removal only	25%
					Placement of plate 2.5	25%
					Plate removal, placement of plate 2.4	25%
					Plate removal, custom-made prosthesis positioning	25%
Plate dislocation	1	–	–	6 years	Plate removal, placement of custom-made prosthesis	100%

* One patient had two events of plate fracture. The total number of patients with complications in the table is 22 while in the text is 14. This is because some patients showed multiple complications as can be detected in Table 2.

Plate fracture occurred in four cases. One of these just had the fractured plate removed with no additional correction. One underwent 2.5 bridging plate repositioning after 2.4 fractured plate removal. One patient had this complication twice (it was considered as two separate complications in Table 3). In the first operation, the broken 2.4 plate was replaced by another bridging plate; however, in the second surgery, it was replaced with a custom-made plate that was fixed to the bone to reconstruct the gap.

Plate dislocation was experienced only in one case, after 6 years from the first surgery. Also in this case the plate was removed, and a custom-made plate was applied in its place.

Two of the 34 patients went through sarco-radionecrosis after 10 months. Both underwent adjuvant radiotherapy and plate removal, and necrotic bone resection was performed. In one case, primary closure was performed without the placement of any plates, due to the patient's poor clinical condition, which did not allow for a more complex surgical procedure. In another case, a custom-made prosthesis was applied, and the pectoralis major pedicled flap was raised to cover the soft tissue defect. One year after the custom-made plate operation, there have been no complications, the occlusion was good, and patients were satisfied with the result, in all three cases.

Recurrence occurred in 14 patients (41%); the average time of recurrence was 15 months (from 1 month to 6 years). Eight cases of local recurrence, of whom three patients had lymph node metastasis and three patients developed distant metastasis involving the bone, liver, and lung. Palliation was the most common accompanying therapy (nine cases). One patient received radiotherapy in combination with chemotherapy and one chemotherapy alone. In one case, contralateral hemimandibulectomy and fibula flap were performed because of neoplastic recurrence of the contralateral side. In this case, a fibula flap was selected because the patient had previously undergone radiation therapy and presented with a through-and-through defect at the mandibular symphysis (B5 defect according to the Della Monaca–Valentini classification) (20). Regarding lymph node involvement and the relationship of this parameter with the rate of disease recurrence, the following data emerged. The absence of lymph node involvement was observed in 15 patients, among whom one experienced disease recurrence. Five patients were classified as N1; among these, one developed disease recurrence. Four patients were classified as N2a, and none of them experienced disease recurrence. Four patients were classified as N2b, of whom two developed disease recurrence. Six patients were classified as N3b, of whom three developed disease recurrence. Further details can be found in Tables 2 and 3.

According to the results, the hospitalization period was 7 ± 2 days on average, and the follow-up was scheduled on the 7th day after discharge, on the 14th day, then every 2 weeks for 1 more month, monthly for 3 months, on the 6th month, on the 12th month, and finally with a check-up every year for the following 5 years. Information about patients' dentition, mastication, dietary outcomes, and the potential use of percutaneous endoscopic gastrostomy (PEG) tubes: In a total of 34 patients, 20 had partial edentulism, 7 were rehabilitated with removable denture, 14 were completely edentulous, 8 were rehabilitated with removable total denture, 4 rehabilitated with implant-supported overdenture type prosthesis, and 2 with fixed implant-supported Toronto prosthesis (before surgery). In the postoperative period, the 7 patients with dentures retained the same prosthesis, and teeth that were avulsed for surgical reasons were replaced with dentures. All the others, following surgery, had modifications in their denture to readapt the new structural situation of the oral cavity. Four patients had occlusal obturators placed. The patients with overdenture rehabilitations switched to removable total dentures, and the two patients who had Toronto prosthesis had some strategic implants preserved for a new prosthesis with bar overdenture configuration. For nutrition, all patients had a nasogastric tube for an average of 7 ± 3 days. The PEG tube was used only in four patients who died shortly after, not being able to undergo any additional surgery.

## 4 Discussion

In oncological patients with maxillofacial tumors, accurate planning is critical for achieving a satisfactory result in terms of reconstruction adequacy and volume correction without damaging critical structures (23). Currently, following resection surgery, the options for mandibular reconstruction include osteocutaneous vascularized flaps or alloplastic implants in combination with soft tissue free flaps (3, 6). Mostly, an osteocutaneous free flap is the most preferred option, as it represents the most reliable option for the reconstruction of large mandibular defects in one stage (3, 4, 6). In the literature, there are various options for mandibular reconstructions with autogenous flaps and alloplastic/titanium implants showing different advantages and disadvantages (Table 4). Among alternatives, ALT flaps with titanium plates are reserved for selected cases with severe oncological prognosis and unsuccessful surgeries (24). Modern reconstruction plates provide an osseointegration mechanism at the bone-screw interface and a locking mechanism at the screw-plate interface, resulting in superior hardware stability and fewer complications (4, 25). Titanium alloy properties of these types of plates guarantee specific characteristics of the plate: excellent biointegration, high mechanical strength, radiopacity, and low risk of infections (26–29). According to the outcomes of this research, reconstruction with ALT flap + customized plate for geriatric patients seems feasible whenever other options are not applicable due to various reasons mostly related to the patient-specific conditions. In such surgeries, an additional advantage is the possibility of a more rapid recovery consequently with a significant reduction of hospitalization period. There is an overall decrease in operating times (for fibula flap, isolation of the pedicle and its liberalization, the insetting with microsurgical anastomoses, the average time in our department is approximately 4 h. While, the setup of the ALT free flap, which involves the same phases, takes an average time of approximately 2 h and 30 min) and reduction of hospitalization times in the intensive care unit (by an average of 1 day for ALT flap, in contrast to the 2–3 days normally required for the free fibula flap).

**Table 4:** Comparison of advantages and disadvantages of different types of flaps for mandibular reconstruction.

Type of flap	Advantages	Disadvantages
Osteocutaneus free fibula flap	Gold standard, possibility of rehabilitating the patient with implant-supported prostheses, greater resistance to radiotherapy, load-sharing function	Time-consuming in the operating room, greater morbidity, and greater recovery time for the patient
ALT + standard plate	Second-line surgery, shorter operating time, quicker patient recovery, less morbidity than free fibula flap	Less resistance to radiotherapy, prosthetic rehabilitation on implants is not possible, greater risk of plaque exposure and associated complications, no load-bearing function of the plaque
ALT + custom-made plate	The same advantages as the ALT + standard plate, with the difference that the plate, being customized, is less bulky and adapts perfectly to the defect, further reduction in surgical time, and the possibility of configuring it with multiunit abutment abutments for prosthetic rehabilitation.	The same disadvantages as ALT + standard plate, with the addition of a higher cost, in case of lack of precision or poor correspondence between project and defect it becomes unusable
Scapula flap	Speed of execution, shorter operating time, rescue surgery	Third-line surgery or rescue surgery, poor quantity and quality of bone for possible prosthetic rehabilitation with dental implants, lower resistance to possible radiotherapy, limited for defects that are small in dimensions, greater morbidity compared with ALT, longer recovery time for the patient

In the literature, following demolition and reconstructive surgery in cancer patients, the complication rates seem to be directly proportional to the size of the defect, which is more frequent if the reconstruction involves the anterior portion of the mandible (3, 30, 31). The complication rate, on the other hand, is reduced if this technique is used for selected cases such as composite defects of the lateral mandible and oropharynx, when the soft tissue resection included the base of the tongue, lateral oropharyngeal wall and tonsillar fossa, and soft palate (3, 30). Placing a titanium plate together with a free flap, in a patient with a large mandibular defect, exposes a high risk of failure and results in an unsuccessful outcome as placing an osteocutaneous flap later, in a second operation, in a harvested surgical area (3, 4, 6, 11, 12, 25, 29). Despite this, the complication rate remains high, and the choice of using a free skin flap and reconstruction plate often depends on the conditions of the patient whenever other options are not feasible (such as vascular problems in the leg for fibular flaps). However, patient-specific anatomical and systemic factors can affect the incidence of occurrence of plate-related complications. One of the most important risk factors is radiotherapy, chemotherapy, and nicotine abuse, which weaken the mucosa overlying the plaque (5, 13). The data from this study revealed that 29%, of the patients experienced plate-related complications, which was in line with findings reported in the literature (3, 31). The most common complications were loose osteosynthesis screws, fractures of the reconstruction plate, and extra/intraoral exposure (3, 11, 12, 14, 16).

As reported by various authors, plate exposure remains the most frequent complication associated with the use of plates (6, 26). Some studies suppose that plate exposure is due to the wound contracting while the plate continues to press on the overlying bone and its incidence can be reduced by an appropriate wrapping with ALT flaps (32). Boyd et al. reported intraoral plaque exposure among patients with anterior mandibulectomy defects with problems in jaw opening muscles, denervation of the lower lip muscles, and ptosis of the lower lip (28, 29). In this retrospective study, only 3 out of 34 patients underwent anterior mandibulectomy. Of these, one had plaque exposure as a complication. According to the results, plaque fracture was the second most common complication, which was followed by plate breakage and loss of the screws that may result from stresses either by forces or by fatigue (28). As a prevention, shear and pull-out forces on the screw can be reduced by improving the bone-prosthesis interface to reduce the chance of screw loosening and failure (18). Less common complications were osteoradionecrosis and plate dislocation as reported in the literature (33, 34). In cases of osteoradionecrosis, the use of free flaps as a therapeutic option is limited to selected cases (35). The main challenges associated with the use of free flaps in these patients include skin alterations resulting from radiation therapy (such as increased thickness and fibrosis) and the potential unavailability of ipsilateral recipient vessels due to prior neck dissection or vascular damage caused by radiotherapy (35).

In cases of sarco-radionecrosis, it is preferable to use a pectoralis major myocutaneous flap, possibly in combination with a reconstruction plate if the anterior portion of the mandible is included in the resection, especially in medication-related necrosis of the jaws (MRONJ) patients (36). One of the two cases of osteoradionecrosis reported in this study was treated with the placement of a custom-made prosthesis combined with a pedicled pectoralis muscle flap to restore soft tissue continuity. The pectoralis major flap is a regional flap that can provide a reliable solution for soft tissue deficiency, even in cases where free flap reconstruction is limited by vascular depleted neck (37). In this work, following initial reconstruction surgery with a plate and ALT free flap, as well as subsequent radiotherapy, a specific case involved a patient who developed a recurrence of the disease in the contralateral hemimandible. The affected area included the symphyseal region, with full-thickness involvement. In this instance, the final surgical approach was decided using a fibular osteocutaneous flap. Particularly in patients who have previously received radiotherapy and present with a through-and-through defect of the mandibular symphysis, the use of a titanium reconstruction plate is not viable; therefore, a fibular flap was selected for this reason. Furthermore, for most situations and whenever possible, the free fibula flap seems to be the first choice for the reconstruction of anterior mandibular defects (38).

Complications are risks for long-term success and cause patient discomfort which might necessitate additional surgical procedures (39). According to the opinion of the authors of this research, in case of the need for additional surgery due to complications following ALT free flap in combination with a reconstruction plate, when possible, it would be better to prefer the placement of a custom-made prosthesis, rather than conventional ones, because of better mechanical characteristics. In cases when a soft tissue deficiency coexists, it would be better to prefer the use of a pedicled flap to bridge the gap, such as a pedicled major pectoralis flap, to reduce microvascular risks, especially for patients who have already had flap failure for vascular circulatory-related reasons. In selected patients, the use of customized plates can be a viable alternative to traditional microsurgical techniques or the use of reconstruction plates, either as a first or second surgery strategy following complications. Custom-made plates as a surgical strategy have the advantage of respecting the patient's anatomy, providing aesthetic results, and reducing the operation time and the hospitalization time, since no donor region can create an additional risk of a complication for the patient. However, in such cases, another problematic issue can arise as it can be very difficult to achieve dental rehabilitation (3, 29, 39).

According to the results and experiences of the authors of this work, when complications are evaluated, the dehiscence mostly occurs within 4 days after surgery due to the development of tension at the suture level as an initial phase of edematous imbibition of the tissues which have obviously lost their normal lymphatic drainage. This may be associated with the exposure of plaque which, being inert, can become contaminated given that the oral cavity is not aseptic. The subsequent infection state can create abscesses which evolve with the development of fistulas and phlegmons. In both cases, if there is neck involvement, this can compromise the vascular anastomoses and subsequently trigger thrombotic complications with or without associated phlebitis, compromising the vascularization and therefore the vitality of the flap. In this pathophysiological mechanism, the time for development is not always predictable because the underlying conditions are multifactorial, one over all the general systemic conditions and associated comorbidities. Vasospasms and thromboembolic complications that can affect the anastomoses can trigger necrosis of the flap, resulting in detachment of the insetting and subsequent exposure of the plaque which can become contaminated in the same way described above. Vascular complications can generally develop within 3 weeks, with high risk in the first and then with a gradual reduction in the following 2 weeks. In the literature, a high incidence of plate fractures is associated with the 2.4/2.5 mm reconstruction plates. But in this work, these thicknesses were preferred, instead of stronger 3 mm plates. In our opinion, the high incidence of fractures is not a result of the thickness, because a thickness of 2.4/2.5 mm is sufficient for the plate to perform its load-bearing function. Fractures seem to develop mostly after loosening of some fixing screws. Under such conditions, the plate is unable to support the load, and therefore, regardless of its thickness, it might fracture. Therefore, a plate with a greater thickness, for example, 3 mm, will not be feasible to reduce fractures, with the fact that it would be even more bulky and would force the use of longer fixing screws which could damage vessels and nerves. However, this statement should be confirmed by further scientific research.

The choice of the material for custom-made plates is another important point (40, 41). In the literature, a wide range of alloplastic implant materials have been used to correct facial asymmetries, defects, and deformities. Among the most used materials are titanium, porous polyethylene (Medpor), polyether ether ketone (PEEK), silicone, and polymethyl methacrylate (PMMA). Each material possesses different physicochemical properties and is associated with a variety of advantages and risks (40). Traditionally, the gold standard material for the reconstruction of the jaw is titanium, because of its biocompatibility, absence of foreign body reaction, and ability to withstand mastication forces. According to a very recent systematic review, PEEK, titanium, and polyethylene implants have successful results in terms of esthetics. However, when implant performance was evaluated for biocompatibility, safety profile, and patient satisfaction, none of the materials was able to stand out among others (40). The results showed that titanium and PEEK implants are mostly preferred when structural rigidity is important. Porous polyethylene is chosen due to its low complication rate and ease of handling, since the lowest rate of postoperative complications is observed with polyethylene implants (FIs). However, the lack of standardized outcome evaluation and heterogeneity of results still represent a huge limitation in comparing the outcomes and reaching a conclusion. There is still a lack of randomized controlled trials that assess the outcomes of different materials, and this prevents the ability to establish a protocol to understand the causality and the effectiveness of different implant types.

The use of reconstruction plates in combination with ALT flap is justified by the patient's conditions and comorbidities and the cancer stage (39). The primary benefits of such flaps for elderly patients are a reduction in operative time, intensive care unit stay, and an overall decrease in hospitalization period. At the clinic of the University of Verona, the combination of reconstruction plaque and ALT free flap is adopted to bridge the bone and soft tissue gap only in patients with advanced age, poor performance status, advanced stage of disease, and high ASA class. Patient-specific implants (PSIs) or custom-made plates play a key role in virtually guided surgery, especially in mandibular reconstruction (41). They represent successful outcomes, such as more stability, and contribute to the accuracy of the results with the planned. More advantages when compared with conventional plates can be listed as; high flexibility in plate design and screw placement, reduced operating times, and potential biomechanical improvements which can be less prone to plate fatigue fractures (41). Currently, there is still a limited number of reports on the use of custom-made plates to restore mandibular defects in patients who are not candidates for microsurgical flap reconstruction (3, 29, 39).

The limitations of this study include small sample size, the retrospective design, selection bias, heterogeneity in complications/salvage techniques, and surgical treatments performed in a single center with no control group using different materials to compare results.

## 5 Conclusions

In conclusion, although not free of risks, reconstruction with an anterolateral thigh (ALT) free flap in combination with a reconstruction plate can be considered as an alternative in extreme cases in which other gold standard reconstruction procedures are not applicable due to advanced age and poor prognosis. In such cases, the use of customized plates can reduce further complications at the reconstruction site and at a harvested bone region which might encounter regeneration problems. However, further studies are required to confirm this statement with control groups and larger sample sizes.

## Data Availability

The raw data supporting the conclusions of this article will be made available by the authors, without undue reservation.
